# High maternal body condition score in beef cattle: gut microbiota dysbiosis, immune dysregulation and offspring health implications

**DOI:** 10.2478/jvetres-2025-0068

**Published:** 2025-12-10

**Authors:** Liang Chi, Yukun Yue, Rui Cao, Xinxin Zhang, Sishuang Wang, Jingxian Li, Yuhan Bing, Ruiguo Wang, Huanqi Liu

**Affiliations:** College of Veterinary Medicine, Qingdao Agricultural University, Qingdao 266109, China; Technology Department, Qingdao Realvet Bio-Technology Co., Ltd, Qingdao 266111, China; College of Agriculture and Forestry, LinYi University, Linyi 276005, China

**Keywords:** beef cattle, body condition, immune function, intestinal flora, maternal obesity

## Abstract

**Introduction:**

This study investigates the correlation between intestinal flora and body condition score (BCS) in beef cattle, focusing on the impact of maternal body condition on gut microbiota and immune function in both cows and their offspring.

**Material and Methods:**

Faecal and blood samples were collected at various stages before and after parturition from Hereford beef cattle categorised into normal, higher and high BCS groups. Microbial DNA was extracted and analysed using 16S rRNA gene sequencing to assess microbial diversity and community structure.

**Results:**

The research indicated that while maternal BCS had minimal impact on the gut microbiota of cows before and after parturition, significant differences were observed in the microbial composition of calf gut microbiota, particularly those of calves born to cows with higher BCS. Calves from high-BCS cows exhibited increased levels of *Proteobacteria*, a potential marker for dysbiosis. Immune function analysis revealed higher levels of interleukin 6 and tumour necrosis factor α in both cows and calves from higher-BCS groups, suggesting a link between maternal obesity and offspring health risks.

**Conclusion:**

The findings point to the importance of managing body condition in pregnant cows to optimise calf health and reduce disease risks and to the linked role of gut microbiota.

## Introduction

Improving the efficiency of beef production not only enhances profitability for farmers but also reduces the environmental impact per unit of production. Although genetic selection has made strides in optimising the efficiency of beef cattle, other factors such as diet, environment, management strategies and the host microbiome are equally vital in determining this trait (21). However, dietary management is frequently suboptimal: many beef cattle farms feed excessive protein during pregnancy, pursuing economic gains and rapid growth. This leads to overnutrition and excessively high cow body condition scores (BCS) (4, 6).

Studies have identified several key gut bacterial groups that are essential for maintaining a healthy gut environment (25). The gut microbiota supports nutrient intake, immune regulation and intestinal barrier integrity. The rumen microbiota state is now widely acknowledged as a crucial factor in maintaining intestinal homeostasis, promoting the development of mucosal and lymphoid structures, and activating the host’s immune cell repertoire (13). The diversity and composition of the gut microbiome are closely associated with species and health status (23), and in cattle the intestinal flora can be posited as one non-genetic factor linked to body condition. This is suggested by studies conducted on animal models, which have detected a correlation between changes in intestinal flora and body weight (1, 22).

Visual evaluation of BCS is a simple, cost-effective and reasonably reliable means of estimation of cattle body energy reserves. It was stated that the BCS directly showed the nutritional, health and reproduction status of cattle (17). For instance, a BCS higher than 3.5 was associated with an approximate 2.5-fold increased risk of ketosis compared to this risk in cows with a BCS of 3.25 or lower at calving (8). Furthermore, numerous studies have demonstrated that obesity or high BCS can impair immune function in humans and rodents (14).

The aims of this study were to investigate the characteristics of the gut microbiota in cows with different body conditions and their offspring, and to analyse these animals’ immune function and the calves’ birth weights. The findings provide novel perspectives and a theoretical foundation for optimising beef cattle production and enhancing the health status of calves.

## Material and Methods

### Experimental animals and faecal samples

Hereford beef cattle which were about to calve were provided by Shandong Longmingniu Rizhao Beef Cattle Farming Co., Ltd (Rizhao, China). These cattle were divided into a normal body condition group (Norm) (score 5–6, n = 5), a higher body condition group (Higher) (score 8, n = 5) and a high body condition group (H) (score 9, n = 5) based on their physical condition (9) (Supplementary Table 1). The calves of these cattle also carried the maternal body condition group differentiation, and were Norm-calf (n = 5), Higher-calf (n = 5) and H-calf (n = 5). The birth weights of the calves were recorded. Fresh rectal faeces were collected at 7 d antepartum (–7d), 7 d postpartum (+7d) and 14 d postpartum (+14d). Additionally, fresh rectal faeces from the calves of the cows in each group were also gathered at 7 d (+7d). The samples were placed into sterile cryopreservation tubes, appropriately labelled, rapidly frozen using liquid nitrogen and stored at –80°C. Blood samples of 10 mL volume were collected *via* coccygeal venepuncture from cows at –7d, 1 day postpartum (+1d), and +7d, prior to morning feeding. Calf blood samples of 10 mL volume were obtained *via* jugular venepuncture at +7d.

### Extraction of genome DNA

Total genome DNA from faecal samples was extracted using a cetyltrimethylammonium bromide/sodium dodecyl sulfate method. The concentration and purity of the extracted DNA was monitored on 1% agarose gels. A concentration of 1 ng of DNA per μL was established using sterile water.

### Amplicon generation

A 16S V4–V5 primer pair was used: 515F with the sequence 5′-GTGCCAGCM GCCGCGGTAA-3’ and 907R with the sequence 5′-CCGTCAATTCCTTTGAGTTT-3′. An 18S V9 primer pair was also used: 1380F with the sequence 5′-GCGGTAATTCCAGCTCCAA-3′ and 1510R with the sequence 5′-AATCCRAGAATTTCACCTCT-3′. The final primer pair used was for internal transcribed spacers (ITS): ITS1F with the sequence 5′-GATGAA GAACGYAGYRAA-3′ and ITS2R with the sequence 5′-TCCTCCGCTTATTGATATGC-3′. All PCR reactions were carried out in 30 μL reaction mixtures with 15 μL of Phusion High-Fidelity PCR Master Mix (New England Biolabs, Ipswich, MA, USA), 0.2 μM of forward and reverse primers and approximately 10 ng of template DNA. Thermal cycling consisted of initial denaturation at 98°C for 1 min; 30 cycles of denaturation at 98°C for 10 s, annealing at 50°C for 30 s, and elongation at 72°C for 60 s; and a final elongation step at 72°C for 5 min.

### PCR product quantification and qualification

The products were mixed with an equal volume of 1× loading buffer (containing SYBR Green) and subjected to electrophoresis on a 2% agarose gel for detection. Samples with bright main bands between 400 and 450 base pairs (bp) were selected for further analysis.

### PCR product mixing and purification

The products were mixed in equimolar ratios. Then, the mixture of PCR products was purified with a GeneJET Gel Extraction Kit (Thermo Scientific, Vilnius, Lithuania).

### Library preparation and sequencing

Sequencing libraries were generated using Next Ultra DNA Library Prep Kit for Illumina (New England Biolabs) following the manufacturer’s recommendations, and index codes were added. The library quality was assessed on a Qubit@ 2.0 Fluorometer (Thermo Scientific, Singapore) and Agilent Bioanalyzer 2100 system (Agilent, Waldbronn, Germany). Finally, the library was sequenced on a MiSeq platform (Illumina, Shanghai, China) and 250 bp/300 bp paired-end reads were generated.

### Data analysis – paired-end read assemblies

Paired-end reads from the original DNA fragments were merged using the FLASH (fast length adjustment of short reads) tool when at least some of the reads overlapped the read generated from the opposite end of the same DNA fragment (11). Paired-end reads were assigned to each sample according to the unique index codes.

### Data analysis – operational taxonomic unit (OTU) cluster and species annotation

Sequence analyses were performed in the UPARSE software package using the UPARSE-OTU and UPARSE-OTUref algorithms (5). In-house Perl scripts were used to analyse α (within-sample) and β (among-sample) diversity. Sequences with ≥97% similarity were assigned to the same OTUs. A representative sequence was selected for each OTU, and taxonomic annotation was performed using the Ribosomal Database Project classifier (12). Alpha diversity was evaluated by rarefying the OTU table and calculating three metrics: Chao1 estimated species richness, observed species counted unique OTUs per sample and the Shannon index measured species diversity (accounting for richness and evenness). Rarefaction curves were generated based on these metrics.

### Data analysis – α diversity

Alpha diversity indices, including Simpson and Chao1, were calculated using QIIME (quantitative insights into microbial ecology) software package v1.9.1 (2). Rank abundance curves and rarefaction curves for OTUs were generated using the ggplot2 package (v2.2.1) within the R software environment. Between-group comparisons of alpha diversity indices were performed using Welch's *t*-test and the Wilcoxon rank-sum test implemented in the Vegan package (v2.5.3) for R. Among-group comparisons of α diversity indices were conducted using Tukey’s honestly significant difference test and the Kruskal–Wallis H test in the Vegan package (v2.5.3).

### Data analysis – phylogenetic distance and community distribution

Graphic representation of the relative abundance of bacterial diversity from phylum to species was created in Krona charts. Cluster analysis was preceded by principal component analysis, which was applied to reduce the dimension of the original variables using QIIME. The package calculates both weighted and unweighted UniFrac distances, which are phylogenetic measures of β diversity. Unweighted UniFrac distances were used for principal coordinate analysis (PCoA) and unweighted pair group method with arithmetic mean (UPGMA) clustering. The obtention and visualisation of principal coordinates from complex, multidimensional data is possible with PCoA. It takes a transformation from a distance matrix to a new set of orthogonal axes, by which the maximum variation factor is demonstrated by the first principal coordinate, the second maximum one by the second principal coordinate, and subsequent variations are shown by the remaining coordinates. Hierarchical clustering is the purpose of UPGMA, which uses average linkage to interpret the distance matrix. Predicted functional profiles of microbial communities were generated using the phylogenetic investigation of communities by reconstruction of unobserved states (PICRUSt) technique. These predicted functional composition profiles were then aggregated into Kyoto Encyclopedia of Genes and Genomes (KEGG) orthology (KO) level 3 pathways. Differences in bacterial functional profiles were assessed using Student’s *t*-test.

### Determination of the serum immune factors interleukin 6 and tumour necrosis factor α

ELISA kits (Shanghai Epizyme Biomedical Technology, Shanghai, China) for interleukin 6 and tumour necrosis factor α were equilibrated at room temperature for 15–20 min after removal from cold storage. The assays were carried out in accordance with the kits’ instructions. After completing the assay protocol, 50 μL of stop solution was added to each well (a colour change from blue to yellow was observed immediately). Within 15 min of stop solution being added, a microplate reader was used to measure the absorbance (optical density value) of each well at 450 nm. The resulting data were subjected to further statistical analysis.

### Statistical analysis

To confirm differences in the abundances of individual taxa between the groups, Metastats software was utilised (16). Linear discriminant analysis effect size (LEfSe) was used for the quantitative analysis of biomarkers within different groups (21). This method was designed to analyse data in which the number of species is much higher than the number of samples and to provide biological class explanations to establish statistical significance, biological consistency and effect-size estimation of predicted biomarkers. To identify differences in microbial communities between the groups, analysis of similarities (15, 16) were performed based on the Bray–Curtis dissimilarity matrices. ELISA results were given as mean ± standard deviation. Data were compared by one-way analysis of variance followed by Tukey’s test when significant differences were found at the P-value ≤ 0.05 level. Statistics were analysed using SPSS 22.0 software (IBM, Armonk, NY, USA). All assays were carried out in triplicate independently.

## Results

### Microbial biomass and community structure in antepartum dams

**Fig 1.**
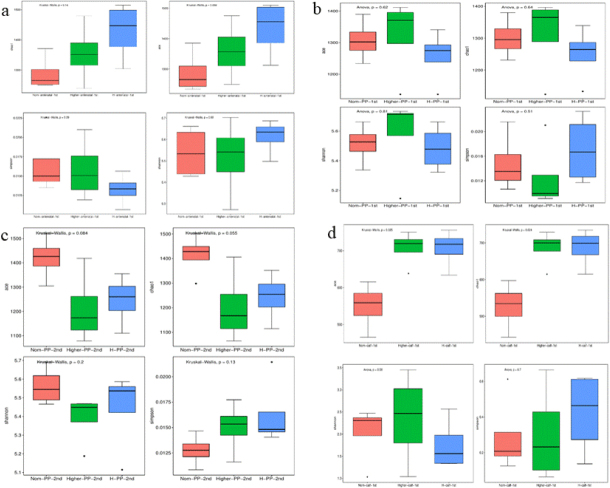
Changes in gut microbiota α diversity in faecal samples of Chinese Hereford beef dams and calves. a) α diversity (ACE, Chao1, Shannon and Simpson indices) of faecal bacterial communities in preparturient dams (-7d) of different body conditions; b) α diversity of faecal bacterial communities in postparturient dams (+7d) of different body conditions; c) α diversity of faecal bacterial communities in postparturient dams (+14d) of different body conditions; d) α diversity of faecal bacterial communities in calves (+7d). Norm antenatal 1^st^ – samples from dams at –7d of normal body condition; Higher antenatal 1^st^ – samples from dams at –7d of higher body condition; H antenatal 1^st^ – samples from dams at –7d of high body condition; Norm PP 1^st^ – samples from dams at +7d of normal body condition; Higher PP 1^st^ – samples from dams at +7d of higher body condition; H PP 1^st^ – samples from dams at +7d of high body condition; Norm PP 2^nd^ – samples from dams at +14d of normal body condition; Higher PP 2^nd^ – samples from dams at +14d of higher body condition; H PP 2^nd^ – samples from dams at +14d of high body condition; Norm calf 1^st^ – samples from calves at +7d born to dams of normal body condition; Higher calf 1^st^ – samples from calves at +7d born to dams of higher body condition; H calf 1^st^ – samples from calves at +7d born to dams of high body condition. Results are presented as mean ± standard deviation. Different letters indicate statistical significance at P-value < 0.05

The ACE and Chao indices reflected the community richness and the Shannon and Simpson indices reflected the community diversity. Differences in gut microbiota bacterial communities between dams of different body conditions were compared at –7d (Fig. 1a). The ACE, Shannon and Chao indices were lower in group Norm than in groups Higher and H; however, the differences between them were not significant. The Simpson index was lower in group H than in the other groups; however, these differences were also not significant. These results indicate that before parturition, there were no significant differences in gut microbiota diversity between cows of different body conditions.

### Microbial biomass and community structure in postpartum dams

The Ace and Chao1 indices derived from analysis of normal condition cow faecal samples were lower than these indices from higher and high body condition cow samples; however, the differences were not significant (Fig. 1b). Furthermore, two weeks after parturition, differences in the cows’ gut microbiota also could be associated with BCS but these differences were not significant (Fig. 1c). The above results indicated that the biodiversity of gut microbiota in cows with different body conditions was susceptible to being influenced by parturition, but the difference in impact was not significant.

### Microbial biomass and community structure in newborn calves

The ACE and Chao1 indices derived from analysis of faecal samples from calves delivered by normal-condition cows were significantly lower than these indices from samples from calves delivered by higher- and high-body-condition cows (Fig. 1d). This finding suggested that the body condition of cows may significantly influence the intestinal microbiota composition in their offspring.

### PCA analysis

**Fig 2.**
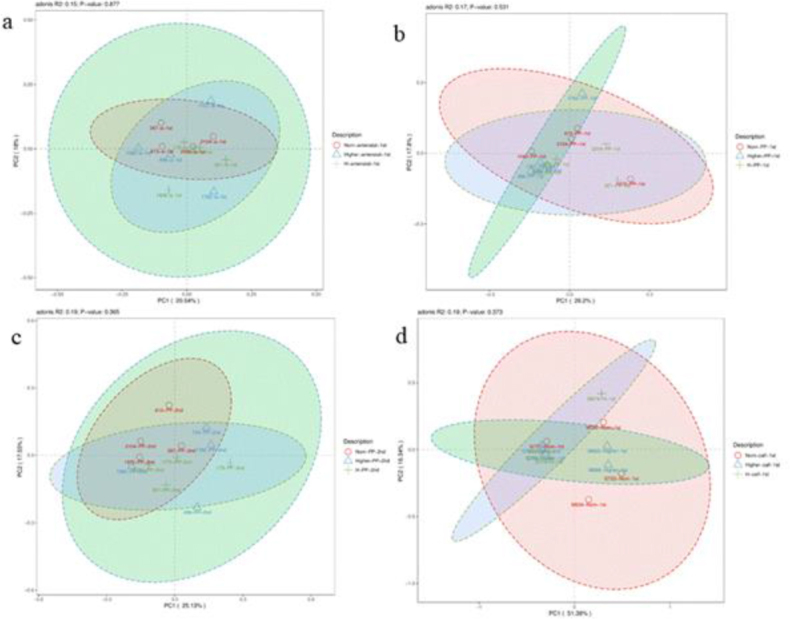
Principal coordinate analysis (PCoA) of gut microbiota in faecal samples of Chinese Hereford beef dams and calves. a) PCoA of faecal bacterial communities in preparturient dams (-7d) of different body conditions; b) PCoA of faecal bacterial communities in postparturient dams (+7d) of different body conditions; c) PCoA of faecal bacterial communities in postparturient dams (+14d) of different body conditions; d) PCoA of faecal bacterial communities in calves (+7d). Norm antenatal 1^st^ – samples from dams at –7d of normal body condition; Higher antenatal 1^st^ – samples from dams at –7d of higher body condition; H antenatal 1^st^ – samples from dams at –7d of high body condition; Norm PP 1^st^ – samples from dams at +7d of normal body condition; Higher PP 1^st^ – samples from dams at +7d of higher body condition; H PP 1^st^ – samples from dams at +7d of high body condition; Norm PP 2^nd^ – samples from dams at +14d of normal body condition; Higher PP 2^nd^ – samples from dams at +14d of higher body condition; H PP 2^nd^ – samples from dams at +14d of high body condition; Norm calf 1^st^ – samples from calves at +7d born to dams of normal body condition; Higher calf 1^st^ – samples from calves at +7d born to dams of higher body condition; H calf 1^st^ – samples from calves at +7d born to dams of high body condition

**Fig. 3.**
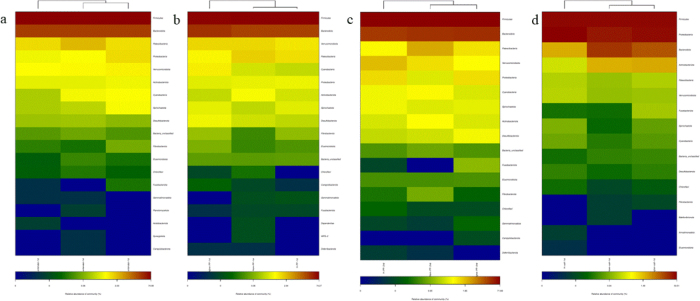
Heatmap of gut microbiota relative abundance at the phylum level in faecal samples of Chinese Hereford beef dams and calves. The colour scale represents the relative abundance of operational taxonomic units. a) Heatmap of faecal bacterial community in preparturient dams (-7d) of different body conditions; b) Heatmap of faecal bacterial community in postparturient dams (+7d) of different body conditions; c) Heatmap of faecal bacterial community in postparturient dams (+14d) of different body conditions; d) Heatmap of faecal bacterial community in calves (+7d). Norm antenatal 1^st^ – samples from dams at –7d of normal body condition; Higher antenatal 1^st^ – samples from dams at –7d of higher body condition; H antenatal 1^st^ – samples from dams at –7d of high body condition; Norm PP 1^st^ – samples from dams at +7d of normal body condition; Higher PP 1^st^ – samples from dams at +7d of higher body condition; H PP 1^st^ – samples from dams at +7d of high body condition; Norm PP 2^nd^ – samples from dams at +14d of normal body condition; Higher PP 2^nd^ – samples from dams at +14d of higher body condition; H PP 2^nd^ – samples from dams at +14d of high body condition; Norm calf 1^st^ – samples from calves at +7d born to dams of normal body condition; Higher calf 1^st^ – samples from calves at +7d born to dams of higher body condition; H calf 1^st^ – samples from calves at +7d born to dams of high body condition

This analysis of OTU levels using the adonis test showed that there was no significant clustering of dams’ samples by body condition groups (Fig. 2a, 2b and 2c). In contrast, calves’ samples did show significant clustering by maternal body condition (Fig. 2d). The top 50 phyla by abundance are shown in Fig. 3. *Firmicutes* was the most dominant phylum in dams and calves. *Bacteroidota* was the second most dominant phylum in dams, while *Proteobacteria* was the second most dominant in calves. These results indicated that there were certain differences in the proportions of intestinal flora species between calves and dams.

### LEfSe analysis

**Fig. 4.**
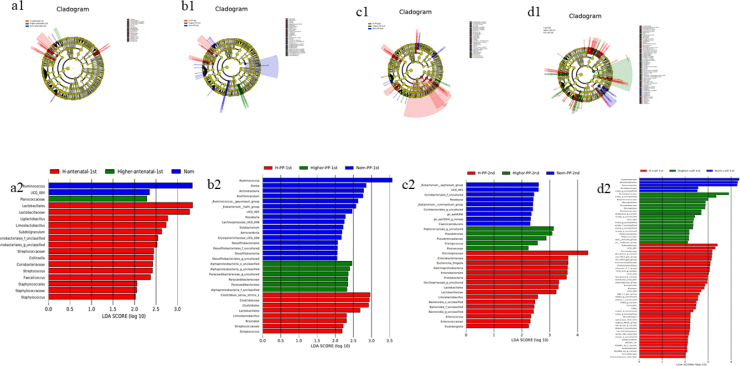
Linear discriminant analysis (LDA) effect size of gut microbiota in faecal samples of Chinese Hereford beef dams and calves. Colours indicate microbial groups significantly enriched in the corresponding group and with a significant effect on the different groups. The higher the LDA score, the greater the influence of species abundance on the different effect. a1) Taxonomic tree of faecal bacterial communities in preparturient dams (-7d) of different body conditions; b1) Taxonomic tree of faecal bacterial communities in postparturient dams (+7d) of different body conditions; c1) Taxonomic tree of faecal bacterial communities in postparturient dams (+14d) of different body conditions; d1) Taxonomic tree of faecal bacterial communities in calves (+7d); a2) LDA discrimination results for preparturient dams (-7d) of different body conditions; b2) LDA discrimination results for postparturient dams (+7d) of different body conditions; c2) LDA discrimination results for postparturient dams (+14d) of different body conditions; d2) LDA discrimination results for calves (+7d). Norm antenatal 1^st^ – samples from dams at –7d of normal body condition; Higher antenatal 1^st^ – samples from dams at –7d of higher body condition; H antenatal 1^st^ – samples from dams at –7d of high body condition; Norm PP 1^st^ – samples from dams at +7d of normal body condition; Higher PP 1^st^ – samples from dams at +7d of higher body condition; H PP 1^st^ – samples from dams at +7d of high body condition; Norm PP 2^nd^ – samples from dams at +14d of normal body condition; Higher PP 2^nd^ – samples from dams at +14d of higher body condition; H PP 2^nd^ – samples from dams at +14d of high body condition; Norm calf 1^st^ – samples from calves at +7d born to dams of normal body condition; Higher calf 1^st^ – samples from calves at +7d born to dams of higher body condition; H calf 1^st^ – samples from calves at +7d born to dams of high body condition

This analysis identified species with linear discriminant analysis scores of >3 as potential biomarkers (Fig. 4). Around parturition, *Ruminococcus* was identified as a key biomarker for maintenance of normal body condition in dams. *Sutterellaceae, Burkholderiales* and *Parasutterella* were potential biomarkers for calves born to dams in the Norm group. Distinct biomarkers were also observed for dams at different production stages: *Lactobacillales* and *Lactobacillaceae* may have marked high body condition in dams antepartum, while *Oscillospiraceae* may have done so for high body condition in dams at 14 d postpartum.

### Venn analysis

**Fig. 5.**
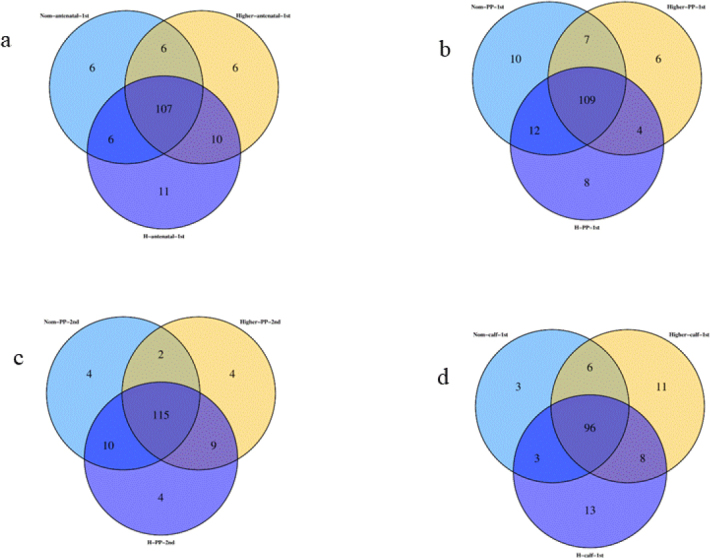
Venn diagrams of shared and unique operational taxonomic units (OTUs) in gut microbiota in faecal samples of Chinese Hereford beef dams and calves. a) Number of shared and unique OTUs in preparturient dams (-7d) of different body conditions; b) Number of shared and unique OTUs in postparturient dams (+7d) of different body conditions; c) Number of shared and unique OTUs in postparturient dams (+14d) of different body conditions; d) Number of shared and unique OTUs in calves (+7d). Norm antenatal 1^st^ – samples from dams at –7d of normal body condition; Higher antenatal 1^st^ – samples from dams at –7d of higher body condition; H antenatal 1^st^ – samples from dams at –7d of high body condition; Norm PP 1^st^ – samples from dams at +7d of normal body condition; Higher PP 1^st^ – samples from dams at +7d of higher body condition; H PP 1^st^ – samples from dams at +7d of high body condition; Norm PP 2^nd^ – samples from dams at +14d of normal body condition; Higher PP 2^nd^ – samples from dams at +14d of higher body condition; H PP 2^nd^ – samples from dams at+14d of high body condition; Norm calf 1^st^ – samples from calves at +7d born to dams of normal body condition; Higher calf 1^st^ – samples from calves at +7d born to dams of higher body condition; H calf 1^st^ – samples from calves at +7d born to dams of high body condition

Through Venn graph analysis, similarities and differences in microbial composition were found (Fig. 5). Specifically, before parturition, the number of OTUs shared among samples from cows of different body types was 107, which may represent the number of microbial taxa ubiquitous in these environments. Some OTUs were not shared: 6 unique OTUs were identified in samples from cows of normal body type, 6 unique OTUs were found in cows of higher body condition and 11 unique OTUs were present in cows of high body condition. After parturition, the number of shared OTUs among cows of different body types increased slightly. One week after parturition, 109 shared OTUs were observed among cows of different body types, and two weeks after parturition, the number increased to 115. Furthermore, calves born to cows of different body types shared 96 OTUs.

### KEGG analysis

**Fig. 6.**
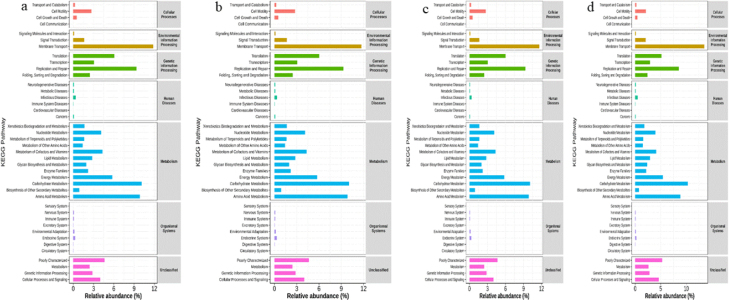
Single-cell transcriptomics analysis and multimodal profiling analysis of functional differences in gut microbiota in faecal samples of Chinese Hereford beef dams and calves based on the Kyoto Encyclopedia of Genes and Genomes (KEGG) database. a) KEGG pathway enrichment in preparturient dams (–7d) of different body conditions; b) KEGG pathway enrichment in postparturient dams (+7d) of different body conditions; c) KEGG pathway enrichment in postparturient dams (+14d) of different body conditions; d) KEGG pathway enrichment in calves (+7d)

This analysis showed that high-abundance bacteria in all body condition groups were significantly enriched in metabolic pathways, particularly carbohydrate metabolism, amino acid metabolism and energy metabolism (Fig. 6).

### The impact of different body conditions of cows on calf birth weight and calf and dam immune function

**Fig.7.**
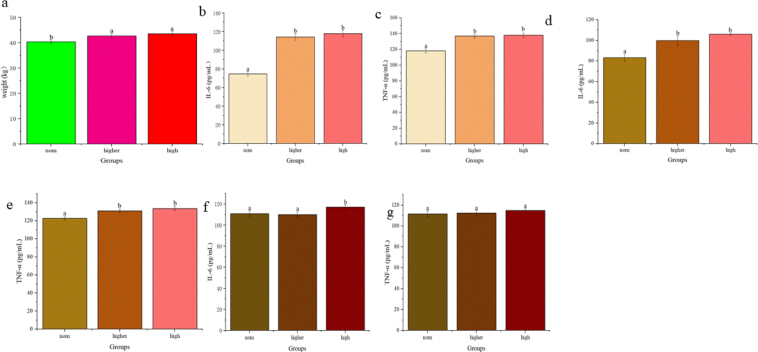
Impact of different dam body conditions on calf birth weight and dam and calf serum levels of interleukin 6 (IL-6) and tumour necrosis factor α (TNF-α) immune factor in blood samples of Chinese Hereford beef stock. a) Birth weight of calves from dams of different body conditions; b) Serum IL-6 level in preparturient dams (–7d) of different body conditions; c) Serum TNF-α level in preparturient dams (–7d) of different body conditions; d) Serum IL-6 level in postparturient dams (+7d) of different body conditions; e) Serum TNF-α level in postparturient dams (+7d) of different body conditions; f) Serum IL-6 level in calves (+7d); g) Serum TNF-α level in calves (+7d). Different letters indicate statistical significance at P-value < 0.05

Calf birth weights are given in Fig. 7a. Birth weights of calves from the Higher and H groups were significantly greater than those from the Norm group, with no significant difference noted between the Higher and H groups.

Serum levels of IL-6 and TNF-α were significantly higher pre-parturition in the Higher and H group dams than in the Norm group dams (Fig. 7b and 7c). Post-parturition, serum IL-6 and TNF-α levels remained so in the Higher and H groups, with no significant difference between these two (Fig. 7d and 7e). Serum IL-6 levels were significantly higher in calves born to dams in the Higher and H groups than in those born to dams in the Norm group (Fig. 7f). However, no significant differences in serum TNF-α levels were observed between calves from different groups (Fig. 7g).

## Discussion

Growing evidence indicates that obesity in mothers before and during pregnancy is associated with a higher risk of obesity and other health conditions in human offspring (7). However, in ruminants, such research is still relatively scarce. In this study, we conducted microbiome analysis on cows with different body conditions and their calves, and analysed immune-related indicators in both the cows and their offspring. We first analysed the microbial biomass and community structure. The results showed that the body condition of cows exerted negligible effects on their microbial biomass and microbiota composition before and after parturition. However, significant differences were observed in the microbial biomass and microbiota structure of calves related to the body condition of their dams. These findings demonstrated that maternal body condition played a role in shaping the microbial abundance and structure in calves, with dams in superior body condition promoting a higher microbial load in their progeny. Principal coordinate analysis based on OTU levels combined with the adonis test revealed that there were no significant differences in the microbiota between cows of different body conditions, but that in the microbiota of calves born to cows with different body conditions there were. The most dominant phylum was *Firmicutes* in all groups, the second dominant phylum in the perinatal stage of adult cows was *Bacteroidota* and the second dominant phylum in calves was *Proteobacteria*. Research indicated that gut microbiota imbalance often arose from a persistent overgrowth of *Proteobacteria*, and that elevated levels of *Proteobacteria* might reliably mark dysbiosis and associated disease risks (19). In this study, we observed a significant increase in the relative abundance of *Proteobacteria* in calves one week postpartum compared to their dams. Furthermore, the proportion of *Proteobacteria* was positively correlated with the body condition score of the dams. These findings suggest that calves born to high-body-condition dams might be at an increased risk of developing various diseases. Therefore, these results highlight the importance of monitoring and managing the body condition of dams to mitigate the potential health risks for their offspring.

Analysis using LEfSe sought to identify biomarker microbiota associated with body condition. The results indicated that around the time of parturition, *Ruminococcus* may be a crucial biomarker for an ongoing normal physiological state. Additionally, *Sutterellaceae, Burkholderiales* and *Parasutterella* could potentially act as biomarkers for normal health condition of the dam when measured in calves. The biomarkers for cow body condition were different at different parts of the periparturient period. *Lactobacillales* and *Lactobacillaceae* may indicate high body condition prior to delivery, *Oscillospiraceae* can be posited to signify high body condition two weeks post-delivery. *Ruminococcus* abundance in the human gut microbiota varies depending on the health status of the host and plays a key role in the modulation of the host immune response (3, 10). *Lactobacillales* was positively correlated with murine intestinal immune-related factors (20). The present observations regarding these taxa in beef cattle faecal samples may indicate that cows with normal body condition have better health status and immune function compared with those with higher body condition.

Based on the aforementioned analyses integrated with microbiome investigations, we hypothesise that cows with elevated body condition scores exhibit different gut microbiota profiles to those of their normal-condition counterparts. Notably, calves born to high-body-condition dams demonstrated a significantly lower proportion of health-associated microbial taxa relative to those born to normal-body-condition dams, suggesting potential maternal transmission of dysbiosis patterns. The significant differences in some of these metabolism-related and immune-related functional microbiota, combined with the detected levels of immune indicators, suggested that the health status of calves born to high-body-condition cows may be inferior to that of calves from cows with normal body condition. Therefore, special attention should be paid to body condition management of pregnant beef cattle. Overfeeding to enhance nutrition for pregnant cows should be avoided, as it may lead to obesity in cows, and negatively impact calf health and complicate their rearing.

## Conclusion

This study clarifies the significant impact of maternal body condition on the gut microbiota composition and immune function of beef cattle and their offspring. Dam gut microbiota showed minimal variation by body condition group, whereas calf gut microbiota showed significant variation by maternal body condition group. The dysbiosis in calves from high-body-condition cows was characterised by an increased relative abundance of *Proteobacteria*, a taxon commonly associated with microbial imbalance and gastrointestinal dysfunction. Additionally, both high-body-condition dams and their calves had elevated serum concentrations of pro-inflammatory cytokines, suggesting a potential transgenerational link between maternal obesity and offspring immune dysregulation. These findings highlight the need for managing the maternal body condition in pregnant beef cattle to foster a healthier gut microbiome and enhanced immune profile in calves. Strategies specifically targeting the prevention of overnutrition and excessive body conditioning during gestation may effectively mitigate the risk of dysbiosis and immune-mediated disorders in offspring, thereby improving calf health and bolstering resistance to disease. This research delivers practical insights for enhancing beef cattle production practices, emphasising the importance of optimal nutrition and diligent monitoring of maternal body condition throughout pregnancy to ensure sustainable herd health and productivity.

## Supplementary Material

Supplementary Material Details
