# Supplementary material for: High maternal body condition score in beef cattle: gut microbiota dysbiosis, immune dysregulation and offspring health implications
Source: J Vet Res. 2025 Dec 10;69(4):617–25. doi: 10.2478/jvetres-2025-0068 (PMC12767147; doi:10.2478/jvetres-2025-0068)
Supplement: Supplementary file 1 — Supplementary Material Details [file jvetres-2025-0068_SM.pdf]

Supplementary Table 1. Standardised bovine body condition scoring for beef cattle

Score	Description	Observations
1	Emaciated	The cow is extremely emaciated, with no muscle felt over the spinous processes, transverse processes, hip bones or ribs. Protrusion around the tailhead and ribs is highly pronounced.
2	Malnourished	The cow still appears somewhat thin, but protrusion around the tailhead and ribs is less obvious. Individual spinous processes remain sharp and palpable, but some tissue covers the back of the ribs.
3	Thin	Individual ribs remain visible, but they do not feel sharp when touched. Palpable fat is evident around the spinous processes and tailhead. Partial tissue covers the back of the ribs.
4	Borderline	Individual ribs are no longer clearly visible. Each spinous process can be palpated but feels rounded rather than sharp. Fat tissue partially covers the ribs, transverse processes and hip bones.
5	Moderate	The cow generally has a good overall appearance, with elastic fat palpable over the ribs and a fat layer detectable around the tailhead.
6	High-moderate	Significant pressure is required to palpate the spinous processes. Abundant fat is palpable around the ribs and tailhead.
7	Good	The cow appears plump, with substantial fat deposits. Elastic fat tissue covers the ribs and tailhead. Fat develops around the vulva and thighs, showing initial signs of weight gain.
8	Fat	The cow is excessively plump and overconditioned. Spinous processes are nearly unpalpable. Large fat deposits surround the ribs and tailhead, and are noted beneath the vulva. “Globular” or “corn cake-like” fat is evident.
9	Overfat	The cow is visibly obese and disproportionate, appearing cumbersome. Thick fat surrounds the tailhead and hip bones, with prominent “globular” or “corn cake-like” deposits. The skeletal frame is no longer visible or palpable. Mobility is severely impaired due to excessive fat accumulation.
